# Recovery time and associated factors of severe acute malnutrition among children in Bahir Dar city, Northwest Ethiopia: an institution based retrospective cohort study

**DOI:** 10.1186/s40795-018-0224-0

**Published:** 2018-04-10

**Authors:** Degnet Teferi Asres, Reddy P. C. J. Prasad, Tadesse Awoke Ayele

**Affiliations:** 1 Department of Applied Human Nutrition, Bahir Dar Institute of Technology, Bahir Dar University, Bahir Dar, Ethiopia; 2 Department of Epidemiology and Biostatistics, Institute of Public Health, College of Medicine and Health sciences, University of Gondar, Gondar, Ethiopia

**Keywords:** Severe acute malnutrition, Treatment outcomes, Recovery time, Northwest Ethiopia

## Abstract

**Background:**

Malnutrition commonly affects all groups in a community, but infants and young children are the most vulnerable. Worldwide, nearly 24 million under five children experience severe acute malnutrition (SAM) which contributes to one million child deaths yearly and 19 million severely wasted children are living in developing countries. While the treatment of severe acute malnutrition (SAM) is well established, achieving desired outcomes has proven to be challenging. There is limited evidence showing the success of treatments in the study area. Therefore, this study aimed to determine recovery time from severe acute malnutrition and identify predictors among children of 6–59 months of age.

**Methods:**

Facility based retrospective cohort study was conducted among 401 children 6–59 months of age who have been treated for SAM. Both descriptive and analytic analyses were *executed*. The results were determined using Kaplan-Meier procedure, log-rank test and Cox-regression. Variables having *P*-value ≤0.2 during binary analysis were entered into multivariate analysis. *P* value < 0.05 was considered as statistically significant.

**Results:**

The recovery rate was 51.9% and the median recovery time was 16 days (95%CI: 14.233–17.767). Controlling for other factors; having anemia at admission, *no plumpy nut provision*, failing to enter in to phase 2 on day 10 and a weight gain of more than 8 g/kg/day were significant predictors of recovery time.

**Conclusions:**

Nutritional recovery rate was far outside of the accepted minimum international standard while median recovery time ranged in the accepted minimum international standard. Children had a lower chance of recovering early when they had anemia at admission, not provided plumpy nut, failed to enter phase 2 on day 10 and failed to gain more than 8 g/kg/day. Therefore, efforts should be strengthened to facilitate early recovery of children by considering the identified predictors of recovery time.

## Background

Malnutrition can be defined as a state of nutrition in which deficiency or excess of energy, protein, and other nutrients causes measurable adverse effects [1]. Although uncommon in developed countries, malnutrition in children remains major public health problem in many developing countries [2]. It continues to be the most important risk factor for growth retardation, illness and death with huge masses of young children particularly affected [3].

Malnutrition can be of the acute, chronic or mixed type [1]. Severe Acute Malnutrition (SAM) is one form of acute malnutrition which refers to weight for height ratio of less than − 3 standard deviations or weight for height ratio of below 70% or Mid Upper Arm Circumference (MUAC) < 110 mm or presence of nutritional edema [4].

Today, 52 million (about 8.3%) children under five in the world are suffering from acute malnutrition. Off those children affected, the majority (> 90%) are found in South and Southeast Asia and sub-Saharan Africa. Despite to communal belief, acute malnutrition (also known as wasting) does not occur only in emergency conditions, it is also common in stable settings in countries like India, Indonesia, Kenya and Ethiopia [5].

Till now, acute malnutrition was also perceived as a condition of humanitarian emergencies rather than a development and public health importance [5]. This is in the face of the long-term economic and social costs associated with the condition. But currently, more attention is started to be given to the problem and recognized as public health and development agenda [5].

While there is a well-established and evidence-based management protocol for treatment of SAM [6, 7], integrating it into essential health packages and achieving desired outcomes has proven to be challenging. This might be due to weaknesses in health systems and challenges in availability of treatment commodities at local levels [8]. Generally, effective management of SAM remains as a huge challenge in low resource healthcare settings [2].

Ethiopia, particularly the study area is not an exception in this regard and the successes of treatment outcomes of SAM in healthcare settings should be tested. However, there are only limited studies on this topic in the country and there was no any study particularly in the study area. The aim of this study was therefore, to determine recovery time from SAM and identify predictors of recovery time among children of 6–59 months of age using the inpatient program at Felegehiwot referral hospital, Bahir Dar city administration, Northwest Ethiopia.

## Methods

### Study area and period

Institution based retrospective cohort study was conducted from March to April, 2016. The study was conducted at Felegehiwot referral hospital which provides inpatient services for the management of SAM in Bahir Dar city administration. Bahir Dar is a capital city of Amhara region and located 565kms from Addis Ababa, North west Ethiopia. The projected population number in 2012 was estimated to be 252,256 of whom 131,930 were females (2011 annual report of Bahir Dar special zone, unpublished). There are 10 government health centers and one government referral hospital in the city administration. There are also private health facilities. Currently, Felegehiwot referral hospital is the only health facility which provides inpatient services for the management of SAM in Bahir Dar city administration.

### Sample size and sampling procedure

All children 6–59 months of age with SAM that have been admitted and treated at therapeutic feeding unit (TFU) of the hospital from October 2012 to April 2016 were eligible for the study. Those children with SAM who did not have proper records (incomplete records/missing) were excluded from the study. Similarly, children with documented secondary undernutrition due to other pathological disorders and with other causes of edema were also excluded from the study. Using Epi info version 6 (CDC, Atlanta) StatCalc programs the sample size was estimated to be 401. Systematic sampling procedure was used to select cases and all cases of SAM were obtained from TFU register book and client cards enrolled from October 1, 2012 to April 30, 2016.

### Operational definitions

Recovery time **–** number of days it takes from admission until a child is recovered from SAM.

Recovered- are those children who have become free from medical complications, edema and have achieved and maintained sufficient weight gain (when they reach 85% weight for length) [9].

Readmission- SAM cases that are declared cured or recovered but relapsed to be admitted (returned back for treatment).

Default- SAM cases that are against (care givers sign on behalf of their child to leave the treatment before the child is cured) or SAM cases that are lost with unknown status.

Phase 2- children are transferred in to phase 2 when they regain good appetite and lose their edema or at least reduced to ++ or + [9].

### Data collection procedures and quality control measures

A structured data abstraction form was used for data collection. Data were gathered for baseline characteristics, routine medications, supplements and therapeutic feedings, follow-up characteristics and outcome status. During data collection, two clinical nurses as data collectors and one public health expert as a supervisor were recruited and 2 d intensive training was given. The data abstraction form was adopted from Ethiopian protocol for the management of severe acute malnutrition [9] and the sphere standard for management of severe acute malnutrition [10].

### Data processing and analysis

Data were entered in to Epi info version 6 statistical software and then exported to SPSS version 20 (IBM, USA) for analysis. Cross tabulation, graph and frequency tables were used to report the descriptive data. Recovery time from SAM was estimated using Kaplan-Meier procedure. Log rank test was used to test whether the observed difference of recovery time between different groups of predictor variables is significant or not. Multivariate Cox proportional hazard regression analysis was carried out to identify predictor variables. Variables having a *P*-value ≤0.2 during binary variable Cox proportional hazard regression analysis were entered into multivariate analysis. *P* value < 0.05 was considered as statistically significant. Both Crude Hazard Ratio (CHR) and Adjusted Hazard Ratio (AHR) with 95% confidence interval (CI) were used to show the strength of association.

## Results

### Sociodemographic and baseline characteristics of children at admission

Out of the total 401 children in the cohort; 195 (48.6%) were males and 206 (51.4%) were females. Majority of study subjects (79.3%) were rural residents. From all cohorts; 95.5% were newly admitted, 40.6% were edematous whereas 56.1% of children were wasted. For almost all children (96.3%) it was not documented whether children passed or failed the appetite test (Table 1).

**Table 1:** Sociodemographic and baseline characteristics of children with severe acute malnutrition, Bahir Dar, Northwest Ethiopia; October, 2012 to April, 2016

Variables		Frequency	Percent
Sex of child	Male	195	48.6
	Female	206	51.4
ᅟResidence	Urban	83	20.7
	Rural	318	79.3
Age of child	6–23	221	55.1
	24–59	180	44.9
Admission status	New	383	95.5
	Return from default	2	.5
	Readmission	16	4.0
Diagnosis at admission	Edematous	163	40.6
	Wasted	225	56.1
	Both	13	3.2
Breastfeeding history	Yes	362	90.3
	No	38	9.5
	Not documented	1	.2
Diarrhea	Yes	211	52.6
	No	190	47.4
Vomiting	Yes	219	54.6
	No	182	45.4
Cough	Yes	205	51.1
	No	196	48.9
Fever	Yes	260	64.8
	No	140	34.9
	Not documented	1	.2
HIV	Yes	21	5.2
	No	155	38.7
	Not documented	225	56.1
Malaria	Yes	15	3.7
	No	382	95.3
	Not documented	4	1.0
Pneumonia	Yes	116	28.9
	No	285	71.1
TB	Yes	38	9.5
	No	360	89.8
	Not documented	3	.7
Anemia	Yes	260	64.8
	No	140	34.9
	Not documented	1	.2
Open skin lesions	Yes	70	17.5
	No	331	82.5
Fever > 39 °C	Yes	45	11.2
	No	356	88.8
Appetite test	Pass	5	1.2
	Fail	10	2.5
	Not documented	386	96.3

### Routine medications

Of all children, more than half (54.6%) received amoxicillin, 67.6% received Ampicilin/gentamicin, 72.8% received measles and only 23.4% of children received de-worming (Table 2).

**Table 2:** Provision of routine medications for children with severe acute malnutrition, Bahir Dar, Northwest Ethiopia; October, 2012 to April, 2016

Variables	Frequency (%)
Amoxicillin	219(54.6)
Ampicilin/Gentamicin	271(67.6)
Deworming	94(23.4)
Measles vaccine	292(72.8)

### Supplements and therapeutic feedings

Out of the 401 children; the majority (82.5%) received folic acid supplement, more than half (58.1%) took vitamin A supplement, almost all (96.0%) received a therapeutic food (F-75) and more than half (54.4%) received plumpy nut. However, it is only 21.2% of children received iron supplement (Table 3).

**Table 3:** Provision of supplements and therapeutic feeding histories of children with severe acute malnutrition, Bahir Dar, Northwest Ethiopia; October, 2012 to April, 2016

Variables		Frequency (%)
Folic acid		331(82.5)
Vitamin A		233(58.1)
F-75		385(96.0)
F-100		285(71.1)
Plumpy nut		218(54.4)
Iron	Yes	85(21.2)
	No	314 (78.3)
	Not documented	2 (.5)

### Follow up characteristics

From 163 children with edema, 20.2% fail to start to lose edema on day 4. One hundred twenty-nine children out of 401 (32.2%) fail to enter in to phase 2 on day 10 and among edematous children for 16.3% of them edema was still present on day 10. Out of total children included for this study, 13.2% failed to gain more than 8 g/kg/day during phase 2 and 10.5% suffered heart failure (Table 4).

**Table 4:** Follow up characteristics of children with SAM, Bahir Dar, Northwest Ethiopia; October, 2012 to April, 2016

Variables	Frequency (%)
Fail to start to lose edema on day 4	33(20.2)
Edema still present on day 10	14(16.3)
Fail to enter in to phase 2 on day 10	129(32.2)
Fail to gain > 5 g/kg/d for 3 successive days	145(36.2)
Heart failure	42(10.5)
Weight gain > 8 g/kg/day	348(86.8)

### Treatment outcomes

**Fig. 1:**
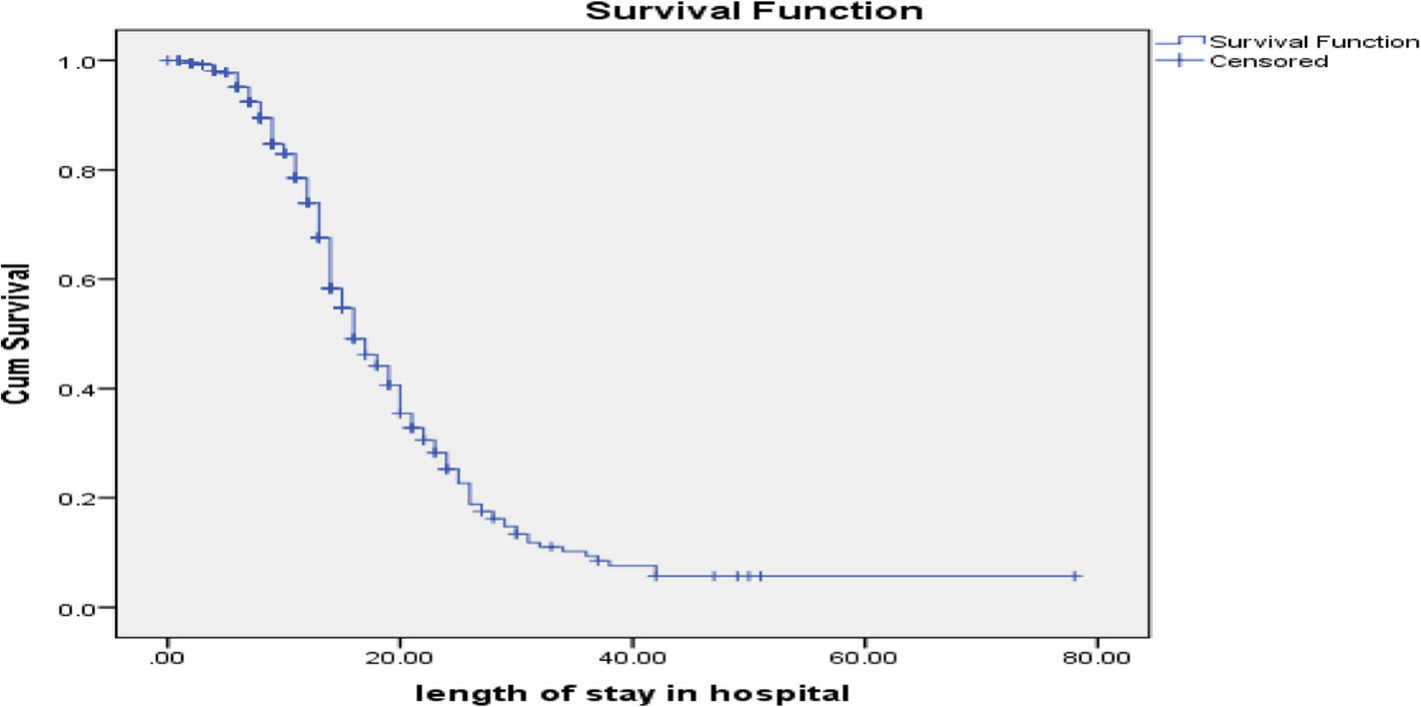
Median recovery time among children with SAM managed at Felegehiwot referral hospital; October, 2012 to April, 2016

Out of the total 401 children in the cohort; 208 (51.9%) were recovered, 17 (4.2%) died, 143 (35.7%) defaulted, 6(1.5%) not responded and 27 (6.7%) transferred (Table 5). The nutritional recovery rate was 2.27 (95% CI: 1.55–3.43) per 100-person day observations among entire subjects in the cohort. The median nutritional recovery time was estimated to be 16 days (IQR: 95% CI; 14.233–17.767) (Fig. 1).

**Table 5:** Treatment outcomes of SAM, Bahir Dar, Northwest Ethiopia; October, 2012 to April, 2016

Variables		Frequency (%)
Status at discharge	Cured	208(51.9)
	Default	143(35.7)
	Died	17(4.2)
	Transfer	27(6.7)
	Non-respondent	6(1.5)

### Predictors of recovery time from severe acute malnutrition

There was a significant difference in the median recovery time between different groups of predictor variables. The median nutritional recovery time for children who had cough at admission was 17 days (95% CI: 14.8–19.2) while it was 15 days (95% CI: 13.3–16.7) for those who didn’t have. Similarly, for children who were HIV positive at admission the median recovery time was 27 days (95% CI: 12.8–41.2) compared to HIV negative which was 17 days (95% CI: 14.1–19.9). The highest difference in median recovery time was observed between children who received plumy nut and those who didn’t, which was 14 days (95%CI: 13.033–14.967) and 28 days (95%CI: 23.301–32.699) respectively (Table 6).

**Table 6:** Kaplan-Meier outputs; recovery time from severe acute malnutrition among children, Bahir Dar, Northwest Ethiopia; October, 2012 to April, 2016

Characteristics		Number (%)	Median recovery time				
			Estimate	95% CI		Log rank X^2^-value	P- value
Cough	Yes	101(49.3)	17.000	14.823	19.177	7.800	.005
	No	107(54.6)	15.000	13.324	16.676		
HIV	Yes	9(42.9)	27.000	12.824	41.176	7.798	.020
	No	90(58.1)	17.000	14.097	19.903		
	Not documented	109(48.4)	16.000	14.552	17.448		
TB	Yes	19(50.0)	26.000	18.096	33.904	18.971	.000
	No	187(51.9)	16.000	14.530	17.470		
	Not documented	2(66.7)	9.000	.	.		
Anemia	Yes	125(60.1)	18.000	16.099	19.901	33.085	.000
	No	83(39.9)	14.000	12.157	15.843		
Fever > 39 °C	Yes	18(40.0)	21.000	13.997	28.003	6.475	.011
	No	190(53.4)	16.000	14.263	17.737		
Plumpy nut	Yes	180(82.6)	14.000	13.033	14.967	34.617	.000
	No	28(15.3)	28.000	23.301	32.699		
Fail to lose edema on day 4	Yes	26(31.3)	24.000	16.471	31.529	4.103	.043
	No	182(57.2)	16.000	14.450	17.550		
Edema still present on day 10	Yes	14(22.6)	24.000	21.447	26.553	4.259	.039
	No	194(57.2)	16.000	14.457	17.543		
Fail to enter phase 2 on day 10	Yes	20(15.5)	30.000	20.971	39.029	46.114	.000
	No	188(69.1)	14.000	13.061	14.939		
Failure to gain more than 5 g/kg/d for 3 successive days	Yes	28(19.3)	26.000	23.262	28.738	28.002	.000
	No	180(70.3)	15.000	14.018	15.982		

Twenty-eight independent variables were analyzed in the Cox proportional hazard regression analysis with the dependent variable. Twelve were significantly associated with recovery time from SAM in the binary Cox proportional hazard regression and 13 variables which have a *p* ≤ 0.2 were entered in to a multiple Cox proportional hazard regression analysis. However, only four variables; being anemic at admission, provision of plumpy nut throughout the treatment, fail to enter in to phase 2 on day 10 and mean weight gain had significant association with the dependent variable. Children who had no anemia at admission were 1.6 times (AHR = 1.552; 95%CI: 1.134, 2.124) more likely to recover earlier compared to those who had. Children who received plumpy nut during their treatment were 2 times (AHR = 2.063; 95%CI: 1.356, 3.139) more likely to recover earlier compared to their counterparts. Similarly, children who entered phase 2 on day 10 were about 3 times (AHR = 2.938; 95%CI: 1.635–5.279) more likely to recover in shorter days compared to those who failed to enter. Children who gained on average more than 8 g/kg/day were 1.2 times (AHR =1.200; 95%CI: 1.014–1.422) more likely to recover earlier compared to those who gained less than 8 g/kg/day (Table 7).

**Table 7:** Predictors of recovery time from severe acute malnutrition among children, Bahir Dar, Northwest Ethiopia; October, 2012 to April, 2016

Covariate		NO. at risk	Cured (N, %)	Crude HR (95% CI)	P-value	Adjusted HR (95% CI)
Cough	Yes	205	101(49.3)	1	.007**	1.073(.799–1.442)
	No	196	107(54.6)	1.455(1.106–1.912)		
HIV	Yes	21	9(42.9)	1	.013**	1.263(.615–2.594)
	No	155	90(58.1)	2.355(1.198–4.628)		
Malaria	Yes	15	5(33.3)	1	.121*	1.184(.504–2.779)
	No	382	202(52.9)	1.904(.844–4.298)		
TB	Yes	38	19(50.0)	1	.000**	1.540(.911–2.603)
	No	360	187(51.9)	2.493(1.529–4.066)		
Anemia	Yes	260	125(60.1)	1	.000**	1
	No	140	83(39.9)	1.713(1.292–2.272)		1.552(1.134–2.124)**
Fever > 39 °C	Yes	45	18(40.0)	1	.015**	1.354(.810–2.262)
	No	356	190(53.4)	1.821(1.121–2.958)		
Plumpy nut	Yes	218	180(82.6)	3.008 (2.019–4.482)	.000**	2.063(1.356–3.139)**
	No	183	28(15.3)	1		1
Fail to start to lose edema on day 4	Yes	17	6(35.3)	1	052*	1.112(.648–1.906)
	No	69	40(57.2)	1.505(.997–2.272)		
Edema still present on day 10	Yes	14	4(22.6)	1	.049**	1.201(.605–2.382)
	No	72	42(57.2)	1.727(1.003–2.974)		
Fail to enter phase 2 on day 10	Yes	129	20(15.5)	1	.000**	1
	No	272	188(69.1)	4.216(2.647–6.714)		2.938(1.635–5.279)**
Failure to > 5 g/kg/d for 3 successive days	Yes	145	28(19.3)	1	.000**	1.229(.771–1.959)
	No	256	180(70.3)	2.716(1.823–4.047)		
Average weight gain	< 8 g/kg/day	53	9(17.0)	1	.025**	1
	> 8 g/kg/day	348	199(57.2)	1.190(1.022–1.386)		1.200(1.014–1.422)**

* Variables have *p*-value ≤0.2 from binary analysis

** Statistically significant (*p*-value <0.05) for both analyses

## Discussion

In this study, the overall recovery time from SAM and differences of recovery time between different groups of children were estimated. The association between recovery time from SAM and independent predictors was also assessed.

Accordingly, the median nutritional recovery times was estimated to be 16 days (95%CI: 14.233–17.767) and it was within the acceptable maximum international standards set at < 28 days [10, 11]. This finding was consistent with other institution-based study in Mekelle, Ethiopia which reported 17 days [12]. However, it was higher than the study done in Zambia that reported 13 days [13]. This might be related to differences in treatment and caring practices, health care settings and other socioeconomic factors among the study areas. Studies indicated that it is only by complying with the standard protocol for management of SAM that better program outcomes could be assured [14].

However, the median nutritional recovery time was lowest compared to the study reports from Kamba District, South West Ethiopia that indicated recovery time of 50 days [15] and Karat and Fasha stabilization Centers, Southern Ethiopia that reported 26 days [16].

Further analysis comparing the median recovery time between different groups of predictor variables showed that there were significant differences in median nutritional recovery time between children with SAM who had cough at admission and those who didn’t have; children with human immunodeficiency virus (HIV) and without HIV and who had tuberculosis (TB) and those who didn’t have. Likewise, there were significant difference in the median recovery time with regard to variables like presence of anemia, fever > 39 °C, provision of plumpy nut, fail to regain appetite on day 4, fail to lose edema on day 4, edema still present on day 10, fail to enter phase-2 on day 10, failure to gain more than 5 g/kg/d for 3 successive days. The highest median recovery time difference was observed between children who received plumy nut and those who didn’t which were 14 days and 28 days respectively.

But, significant differences in median recovery time were not observed between other groups of predictor variables including residence, sex, age, admission status, diagnosis at admission, diarrhea, breastfeeding history (*P*-Value < 0.05). For some predictor variables, this is in consistent with a study conducted in therapeutic feeding centers, Southern Ethiopia, where median recovery time was not significantly different for residence, sex, age [16].

Concerning predictors of recovery time from SAM; presence of anemia at admission, provision of plumpy nut, fail to enter in to phase-2 on day 10 and mean weight gain at discharge had significant association with the dependent variable.

Children who had no anemia at admission were 1.6 times (AHR = 1.552; 95%CI: 1.134, 2.124) more likely to recover earlier compared to those who had. However, a study in Burkina Faso indicated that with a strong respect of current inpatient SAM management, anemia did not have negative impact on nutritional recovery during hospitalization [17] which means even though children are anemic at admission if treated according to the protocol for management of SAM, it doesn’t have negative effect on a nutritional recovery time. For this study, the reason might be due to the fact that children were not receiving iron supplement even when they were anemic. It is because the finding indicated that while 64.8% of severely malnourished children were anemic, it is only 21.2% of the total children enrolled received iron supplement.

Children who received plumpy nut during their treatment were 2 times (AHR = 2.063; 95%CI: 1.356, 3.139) more likely to recover earlier compared to their counterparts. This is in line with a meta-analysis which included 14 studies in low and middle income settings and indicated that children in the ready to use therapeutic food group (RUTF) or plumy nut group were significantly more likely to recover and less likely to be non-responders [18]. According to that meta-analysis, children who received plumpy nut were 1.5 times more likely to recover than those receiving normal therapy. This might be due to the fact that children who received plumy nut might achieve rapid weight gain so that fulfilled the discharge criteria (as cured) early compared to those who didn’t get the chance to consume plumpy nut [16]. A study carried out in Tigray, northern Ethiopia also indicated that plumpy nut had a positive effect to the recovery rate and revealed that as a child consumed one more sachet of plumpy nut, the recovery rate from SAM increased by 4% [14] and this might shorten the recovery time among those who received plumpy nut for this study.

Correspondingly, children who entered in to phase 2 on day 10 were about 3 times (AHR = 2.938; 95%CI: 1.635–5.279) more likely to recover in shorter days compared to those who failed to enter. This might be due to the reason that unless children enter in to phase 2, they will not be given therapeutic foods (F-100 & plumy nut) which can promote weight gain as rapid weight gain at stage one is dangerous. That is why F75 is formulated so that patients do not gain weight during this stage. In Phase-2; they are given plumpy nut or F100. Those formulas are designed for patients to rapidly gain weight (more than 8 g/kg/day). Therefore, children who enter phase 2 early (≤10 days) will enjoy this advantage and recover early compared to their counterparts [16].

Mean weight gain was also significantly associated with median recovery time by which children who gained more than 8 g/kg/day were 1.2 times (AHR = 1.200; 95%CI: 1.014–1.422) more likely to recover earlier compared to those who gained less than 8 g/kg/day. This is consistent with recommendations of the Ethiopian protocol for the management of severe acute malnutrition [9] as well as the sphere standard [10] which state that if children gain more than 8 g/kg/day starting from phase-2 (for wasted children) and after loss of edema (for edematous), the children can recover early within the acceptable minimum standard of days.

### Limitations of the study

As the study was retrospective and based on secondary data, incomplete records were observed in some predictor variables. The research also failed to explore other parental socio-demographic and socioeconomic characteristics.

## Conclusions

The median recovery time ranged in the accepted maximum international standards set for management of SAM. There were significant differences in the median nutritional recovery time between different groups of predictor variables. From the multivariate Cox proportional hazard regression, it was proved that children had a lower chance of recovering early when they had anemia, not provided plumpy nut, failed to enter phase 2 on day 10 and failed to gain more than 8 g/kg/day.

Health care providers (TFU care staff) are strongly advised to comply with inpatient SAM treatment and management protocols in early diagnosis and treatment of anemia, provision of plumpy nut, regular monitoring of weight gain and phase transition. To complement the limitations of this study, further study using prospective design should be conducted for better information including other factors not included under this study such as parental socio-demographic and socioeconomic characteristics, perception of care givers on severe undernutrition and therapeutic feeding programs.

## Abbreviations

AHR: Adjusted Hazard Ratio
CI: Confidence interval
H R: Hazard ratio
MUAC: Mid-Upper –Arm Circumference
OTP: Outpatient Therapeutic Feeding Program
RUTF: Ready to use therapeutic food
SAM: Sever Acute Malnutrition
SD: Standard deviation
SPSS: Statistical Package for Social Sciences
TFU: Therapeutic feeding unit
